# What is in a Mandate? Introducing the UN Peace Mission Mandates Dataset

**DOI:** 10.1177/00220027231159830

**Published:** 2023-03-02

**Authors:** Sara Hellmüller, Xiang-Yun Rosalind Tan, Corinne Bara

**Affiliations:** 1 30525 IHEID, Geneve, Switzerland; 2 Department of Peace and Conflict Research, 8097 Uppsala Universitet, Uppsala, Sweden

**Keywords:** international peacekeeping, mediation, civil wars, conflict management, conflict resolution

## Abstract

UN peace missions are constantly evolving. Yet, we lack a detailed understanding of the shifting types and objectives of peace missions beyond broad categorizations that distinguish for instance between observer, traditional, multidimensional, and peace enforcement missions. To address this gap, we present the UN Peace Mission Mandates (UNPMM) dataset. With global coverage, 30 years of data between 1991 and 2020, a broad scope that includes peacekeeping and political missions, and information on 41 mandate tasks, the UNPMM represents one of the most detailed and up-to-date datasets on UN peace mission mandates. We use it to highlight how mission types, objectives, and specific tasks have changed since the end of the Cold War, and to analyze what factors influence the kind of missions the UN is willing to authorize. The descriptive statistics and empirical analysis reaffirm the need for a greater disaggregation of data on UN peace missions and their mandates.

“*The world is changing and UN peace operations must change with it if they are to remain an indispensable and effective tool in promoting international peace and security*”

Former UN Secretary-General Ban Ki-moon (United Nations 2014)

## Introduction

UN peace missions are constantly evolving.^1^ Observers note changes regarding the *types* of missions established as well as their *objectives*: We are seeing more political missions instead of large peacekeeping operations (Gowan 2010, 4) and a coercive turn wherever peacekeeping operations are deployed, meaning robust conflict management measures involving the use of force to stabilize the security situation and protect civilians (Karlsrud 2019, Curran and Hunt 2020, Duarte Villa and Jenne 2020). Besides these general trends, however, we lack a detailed understanding of the shifting types and objectives of peace missions. This is because existing mission categorizations have remained at an aggregate level, distinguishing for instance between observer, traditional, multidimensional, and peace enforcement missions (e.g., Fortna 2004, Doyle and Sambanis 2006). This means that most peace missions after the end of the Cold War are lumped together under the broad label of multidimensional operations, with the only distinction being whether they are authorized to use force or not. This arguably does not account for the vast diversity of peace missions deployed since 1991 (Hellmüller et al. 2022).

One way of addressing this gap is by analyzing UN peace mission mandates. Mandates give important indications on peace missions’ scope and objectives – they are the missions’ “marching orders” (Security Council Report 2019). This paper presents a novel dataset on UN Peace Mission Mandates (UNPMM).^2^ The UNPMM codes the mandate tasks of all UN peace missions between 1991 and 2020, offering a detailed taxonomy of a total of 41 mandate tasks. Other authors have recently generated disaggregated data on UN peace mission mandates (see Lloyd 2021, Clayton et al. 2021, Di Salvatore et al. 2022). The UNPMM extends the scope of these existing datasets in three ways. First, while existing datasets on peace mission mandates mostly focus on peacekeeping operations (PKOs), the UNPMM also includes political missions, meaning Special Political Missions (SPMs) and Special Envoys/Special Advisors (SE/SAs). This inclusion allows us to analyze dynamics around mission *types*. Second, the UNPMM presents a new inductive categorization of peace missions based on a fine-grained analysis of their individual mandate tasks, distinguishing between minimalist, moderate, and maximalist objectives (Call and Cousens 2007). This addition enables us to explore how mission *objectives* change over time and across contexts. Third, the UNPMM is currently the most spatially and temporally comprehensive dataset. It is global in reach, coding all UN peace missions around the world up until 2020. This gives it an extensive empirical basis, including 113 peace missions that are coded with one of the most detailed coding schemes available, which encompasses 41 different mandate tasks.

Overall, the UNPMM allows for a better understanding of the nature and evolution of UN peace missions since the end of the Cold War. It makes three contributions to the broader study of peace missions. First, it can be used to examine how different factors influence the mandates of UN peace missions. Second, it allows for novel ways to measure mission effectiveness by examining the effect of specific mandate tasks and of their interactions on mission outcomes. Third, it can be used to analyze how individual mandate tasks are adapted and implemented on the ground, thereby contributing to research on local-global interactions.

In what follows, we first make the case for a new mandate dataset by providing an extensive review of existing datasets and we then introduce the UNPMM and its comparative advantages. We subsequently explore patterns in the data showing trends in mission types, objectives, and specific tasks. Third, we show the UNPMM’s potential in a statistical analysis. Specifically, we replicate a study by Cordell et al. (2021) on UN peace mission authorization and add nuance to the authors’ findings by including information on political missions and mandate tasks from the UNPMM. We conclude by summarizing the main contributions of the UNPMM and by suggesting potential qualitative and quantitative insights that can be derived from it.

## The Case for a(nother) Mandate Dataset

The need to overcome broad classifications of UN peace missions has led several scholars to establish more disaggregated datasets that code peace missions’ mandate tasks. An example is the dataset by Di Salvatore et al. (2022) on Peacekeeping Mandates (PEMA) that codes 41 different mandate tasks of 27 UN PKOs in Africa between 1991 and 2017. The Tasks Assigned to Missions in their Mandates (TAMM) dataset established by Lloyd (2021) moves beyond peacekeeping *in Africa*, coding 40 different tasks of 72 UN PKOs globally and extending the time period to include the Cold War period (1948 to 2015).^3^

Both datasets make important contributions to a better understanding of the (shifting) nature of peacekeeping. However, both focus exclusively on PKOs, leaving out political missions. The latter are missions that prioritize “political engagement with governments, parties and civil society aimed at averting, mitigating or stopping conflict” (Gowan 2010, 2). PKOs and political missions are different in that PKOs typically involve military troops and/or police and are assigned security-related tasks (including the delivery of humanitarian assistance), while political missions are usually composed of civilian units and are more focused on political, legal, and economic tasks, even though military and police personnel may be present as guard units to the mission.^4^

The oversight of political missions in existing datasets is not surprising insofar as they have received less scholarly attention than PKOs. As Whitfield (2010, 27) states, “the small size and low price tag of most political missions as compared to peacekeeping operations – as well as the less-than-headline-grabbing nature of many of their achievements – contributes to the relative obscurity of their efforts.” While some SPMs or SE/SAs are analyzed in the mediation literature (Iji 2017, Hudáková 2021, Hellmüller 2021a), studies on PKOs and on political missions remain remarkably segregated.

An exclusive focus on PKOs provides an incomplete picture of UN peace efforts in at least three regards. First, it does not capture larger trends in the deployment of mission types. For instance, since 2014, the UN has only established one PKO (the UN Mission for Justice Support in Haiti from 2017 to 2019) while deploying 11 political missions, suggesting an adaptation rather than demise of peace missions in recent years (Coleman and Williams 2021). Thus, looking only at PKOs does not tell us anything about the conditions and reasons for when the UN chooses one type of peace initiative over another. Second, leaving out political missions may lead to a fragmented assessment of UN peace efforts in a given country. SPMs and SE/SAs can be stand-alone missions, but are often used in conjunction with a PKO to end a conflict or as part of an exit strategy, whereby the PKO is replaced by a new or ongoing political mission (Gowan 2010, Johnstone 2010). Take the case of Burundi: The UN Office in Burundi (UNOB) was initially established as a small SPM with the goal of supporting the government of Burundi in reaching a peace agreement (UN Security Council 1999). However, the worsening security situation led to the closure of UNOB and establishment of a PKO under Chapter VII (ONUB) to stabilize the context and enable the implementation of the Arusha Accords. As the prospects of a negotiated settlement came to the fore, ONUB was terminated and an SPM (BINUB) was established as a means of supporting the transition out of conflict and the withdrawal of UN troops (UN Secretary-General 2006). This shows that focusing exclusively on PKOs does not tell us anything about the interaction and sequencing of different mission types and whether the existence of one type of mission makes the deployment of other types more or less likely. These insights would however be central to the literature on burden-sharing or post-exit effects of PKOs (Bove and Elia 2011, Gledhill 2020, Di Salvatore and Ruggeri 2020, Caplan 2020). Third, looking at PKOs only may lead to a distorted picture of UN peace activities overall. Contexts like Afghanistan, Iraq, or Libya are not studied in the peacekeeping literature, despite the extensive and long-term UN political missions deployed to these countries. Indeed, as Clayton et al. (2021, 164) note, “existing research treats cases without peacekeeping as having seen no UN engagement at all.” For a more accurate account of UN peace missions, it is thus important to extend the analytical framework beyond PKOs.

Clayton et al. (2021) do that in their UN Peace Initiatives (UNPI) dataset, which codes 469 peace initiatives by the UN aimed at conflict prevention and crisis management, mediation, peacekeeping, and peacebuilding between 1946 and 2015. In terms of mission types, they thus go even further than political missions and include a wider set of diplomatic and technocratic initiatives, such as investigative bodies, sanctions monitoring teams, or panels of experts. This breath of initiatives comes, however, at the expense of detail regarding the mandate tasks of each initiative. Indeed, the 27 functions they code to capture what initiatives are tasked to do are relatively broad, such as “peacekeeping operations”, “peacebuilding”, or “implementation” (Clayton et al. 2021).

Besides the inclusion of political missions, the UNPMM extends the scope of existing datasets in two more ways. First, the detailed coding of mandate tasks allows us to make an additional conceptual contribution by adding descriptive labels that distinguish between minimalist, moderate, and maximalist objectives of tasks and missions (Hellmüller et al. 2022).^5^ Minimalist mandate tasks focus on negative peace in that they aim at the absence of violence in the short-term and at structuring the international response. Moderate tasks go beyond a negative peace and include some structural measures that aim at institutionalizing peace. Finally, maximalist tasks aim at a positive peace by addressing the root causes of the conflict through military, political, and socio-economic measures. Existing classifications are either based on the characteristics or themes of tasks rather than their objectives, such as the *monitoring-assisting-securing* distinction in PEMA (Di Salvatore et al. 2022) or the *diplomatic-technocratic-political-development-peacekeeping* distinction in UNPI (Clayton et al. 2021), or they are conceptually ambiguous, such as the descriptive labels *peacekeeping, peacebuilding*, and *violence limitation* in TAMM (Lloyd 2021). Our distinction between minimalist, moderate, and maximalist objectives captures the basic ambition of a task or the mission as a whole on the well-known spectrum from negative to positive peace (Galtung 1969, Call and Cousens 2007). This classification can be useful for instance for studies on peace mission supply. The fact that only a minimalist PKO was deployed to Syria while a maximalist PKO was sent to Mali reflects the different levels of (dis-)unity in the UN Security Council (UNSC) on these two conflicts (Mahapatra 2016). More broadly, the *minimalist-moderate-maximalist* categorization can be used as an indicator of how UN member states perceive the role of the UN in maintaining peace from a restricted to a more intrusive role (Badache et al. 2022). It thus allows for insights that go beyond existing categorizations of mandate tasks and missions.

Second, the UNPMM is currently the most comprehensive and detailed mandate dataset in terms of regional and temporal coverage. It is global in reach, coding all UN peace missions around the world, and it is the most up-to-date, coding missions until 2020.^6^ This gives it an extensive empirical basis including 113 missions.^7^ It also has one of the most detailed coding schemes so far, distinguishing between 41 different mandate tasks.^8^ This allows for novel and detailed analyses of regional and temporal trends in peace missions. We can for instance determine the share of a given mission *type* per region. PKOs dominate most clearly in Europe (mostly due to deployments in the Balkans), the share is equal in Africa and the Americas, and political missions dominate in Asia and the Middle East.^9^ We can also examine the share of a given mission *objective* per region or time period. Regarding the region, maximalist missions dominate in Africa and the Americas, Asia hosts an equal percentage of maximalist and moderate missions, while minimalist missions dominate in the Middle East and Europe.^10^ Regarding the time period, moderate missions were most frequent from 1991 to 2000, maximalist missions dominated from 2001 to 2010, and both minimalist and maximalist missions tied for the most commonly established mission type from 2011 to 2020.^11^ The UNPMM thus allows researchers and policymakers to discern trends and to conduct statistical or qualitative analyses that go beyond what is offered in existing datasets.

## Introducing the UNPMM

The UNPMM codes the mandate tasks of a total of 113 UN peace missions, including 54 PKOs, 50 SPMs, and 9 SE/SAs, that were either established or had mandate changes in the period between 1 January 1991 and 31 December 2020.^12^ The unit of analysis is a mission-year. The majority of missions (more than 60%) do not have any mandate changes after the year of establishment. This may not be surprising, as mandate changes require UNSC approval, but it illustrates why UN operations are at times slow to react to quickly changing circumstances on the ground. The remaining missions, however, occasionally receive new mandate tasks, which is why we provide the data in time-series format. The extreme case is the UN Mission in the DRC (MONUC), which received new tasks in seven of the 11 years during which it was active.

All missions are distinguished by whether they are PKOs or political missions. Within political missions, we further distinguish between SPMs and SE/SAs. SPMs are either regionally- or country-based missions with a political mandate to promote peace, often through multidimensional mandates (Druet 2021, 4). SE/SAs, in turn, are headquarter- or regionally-based high-level mediators with a small accompanying team who implement the good offices role of the Secretary-General (Druet 2021, 4).

Our coding approach involved multiple steps. First, each mission mandate was coded based on the language in UNSC resolutions. If an SPM or SE/SA was authorized by the UN General Assembly (UNGA) or through an exchange of letters between the Secretary-General and the UNSC (Johnstone 2010, 22), we coded on the basis of these letters and UNGA resolutions. When a mission did not have a written mandate (mostly SE/SA), we relied on other UN sources. We coded inductively, starting from the language present in the source, rather than according to pre-conceived categories. We disaggregated tasks as much as possible to allow for the highest granularity. Electoral support, for instance, was divided into electoral assistance and electoral security; Security Sector Reform (SSR) was divided into SSR military and SSR police, or dialogue and reconciliation was distinguished according to the regional, national, or local level. We then consolidated the inductively generated list of tasks within the coding team. To ensure inter-coder reliability, we conducted a second round of coding in which a different person coded each mission. Finally, we validated the coding by analyzing additional UN material as well as secondary literature. If these sources mentioned further tasks, we coded these only if we could locate them in the original UN source. Through this process, we identified a total of 41 mandate tasks listed in Table 1.^13^ We also include a variable that records whether peace missions are authorized to use force in defense of themselves, civilians, and/or the mandate.

**Table 1.** Mandate Tasks in UN Peace Missions, 1991–2020.^14-^16

	Minimalist tasks	Moderate Tasks	Maximalist Tasks
1	Co-ordination of donors, partners, and UN agencies	Conflict assessment and early warning	Civil society capacity building
2	Demilitarization	Disarmament, demobilization and reintegration (DDR)	Dialogue and reconciliation (local)
3	Demining	Electoral assistance	Dialogue and reconciliation (national)
4	Elimination of chemical weapons program	Electoral security	Dialogue and reconciliation (regional)
5	Good offices and mediation (track 1)	Information campaigns	Economic reforms
6	Humanitarian assistance	Monitoring/investigating international humanitarian law (IHL)/international human rights law (IHRL) violations	Good governance
7	Observing, monitoring, or reporting (OMR) military	Recovery, rehabilitation, and reconstruction	Human rights promotion
8	OMR police	Support to international criminal justice	Institution-building (IB) transitional state institutions
9	Refugee/Internally displaced person (IDP) assistance	Protection of civilians (POC) children	Promotion of independent media
10	Small arms and light weapons (SALW)	POC conflict-related sexual violence	Rule of law (ROL) judicial reform
11	Secure environment for delivery of aid	POC general	ROL legal reform
12			ROL penal system reform
13			Sexual and gender-based violence
14			Security sector reform (SSR) military
15			SSR police
16			Support to permanent state institutions
17			Transitional justice
18			Women’s rights and participation
	Ocat: Use of force		

As mentioned above, in addition to this granular coding of mandate tasks, we also include a categorization of tasks and missions that distinguishes between minimalist, moderate, and maximalist objectives (Call and Cousens 2007). However, the dataset can be used with or without this additional categorization. For each task identified, we recorded whether it is mostly aimed at stopping the violence (minimalist), whether it also aims at improving governance (moderate), or whether it aims to address the root causes of conflict and establish positive peace (maximalist). There will always remain a degree of interpretation regarding the assignment of a mandate task to a category. We discuss and justify our rationales in the codebook, especially those that could be considered more controversial.

Beyond individual tasks, we also categorize overall missions as minimalist, moderate, or maximalist. Each mission is assigned a time-constant mission classification that is based on all the mandate tasks established during the mission’s lifespan. To aggregate the minimalist, moderate, and maximalist tasks into an overall mission classification, we apply Goertz’s “best shot principle”, which takes into account the maximum degree of a given feature among disparate entities (Goertz 2006). First, we take minimalist, moderate, and maximalist tasks as comprising three different entities. We then aggregate individual mandate tasks into these three entities, taking the overall objective of a mission to be equivalent with the highest of the three categories into which its tasks fall. For example, if a mission contains 11 minimalist tasks and 1 moderate task, the mission’s overall objective will be moderate. If a mission contains 11 moderate tasks and 1 maximalist task, the mission’s overall objective will be maximalist. We adapt Goertz’s approach to account for two specific situations. The first is where mandates contain minimalist and maximalist tasks only, but the number of minimalist tasks by far outweigh the number of maximalist tasks. In such combinations, the overall mission objective is moderate. The reasoning is that a maximalist classification would not accurately capture the polarization between entities in the relationship. The second situation is where the mission has tasks that are fairly evenly distributed across all three categories. For example, a mission with 1 minimalist, 1 moderate, and 1 maximalist task is classified as moderate. The reasoning is that a maximalist classification would not accurately capture uniformity among entities in the relationship.

According to this aggregation rationale, we determine a mission’s overall score by first assigning a weight of 1 to minimalist tasks, a weight of 2 to moderate tasks, and a weight of 3 to maximalist tasks. We then divide the (weighted) sum of tasks by the overall number of tasks assigned for a mission, resulting in a score between 1 and 3. The formula is as follows:

$$Overall\;Mission\;Score=\frac{\left(\sum \min.\;tasks\times 1\right)+\left(\sum \mathrm{mod}.\;tasks\times 2\right)+\left(\sum \max.\;tasks\times 3\right)}{\sum tasks}$$

Missions with scores equal to 1 are classified as minimalist, missions between 1 and 2 are moderate, and missions with scores above 2 are maximalist. The formula returns an exact mission score that provides more granular information on the exact ranking of a mission. Of course, users who do not share the same understanding of what an “overall” minimalist, moderate, or maximalist mission is can use the detailed information on tasks in the dataset to devise their own formula of aggregation.

The classification of mission objectives is independent of mission types (PKO, SPM, and SE/SA). While there is an empirical relationship between mission type and objectives, as we show further below, any type of mission can have minimalist, moderate, or maximalist objectives.

Our data is easily combined with data by the Uppsala Conflict Data Program (UCDP), or any other data using UCDP conflict IDs. Each mission in the UNPMM is linked to one or several conflict IDs from the UCDP/PRIO Armed Conflict Dataset (ACD), Version 20.1 (Gleditsch et al. 2002, Pettersson and Öberg 2020). To determine the relevant conflict ID, we conducted qualitative research and cross-comparisons between the conflicts referred to in UN and secondary sources and the conflicts listed in the ACD. Moreover, we consulted the UN and Non-UN Peacekeeping Dataset by Bara and Hultman (2020), who also linked peacekeeping operations to UCDP conflicts.

We provide the UNPMM in two versions. The first version is a tabular dataset in mission-year format. The second version is a user interface (UNPMM-UI) developed for both qualitative and quantitative analyses. The UNPMM-UI contains information on, and hyperlinks to, all UN resolutions and sources consulted in the coding of the mandate tasks as well as search functions for easy navigation through the data. Researchers can search mission mandates by abbreviation, start or end date, mission type, mandate tasks, region, or country.

## When to Use Which Dataset

Depending on the purpose of the research, scholars may opt for different datasets of UN peace mission mandates. As mentioned above, the main datasets currently available are the PEMA dataset by Di Salvatore et al. (2022), the TAMM dataset by Lloyd (2021), and the UNPI dataset by Clayton et al. (2021). To guide researchers in the choice of which dataset to use, we provide a detailed overview of how the UNPMM compares to the other datasets in the Annex.

For researchers focusing on PKOs only, the choice between UNPMM, PEMA, and TAMM may be guided by the features or task lists of these datasets. Regarding the features (see Table A1 in the Annex), as specified above, the UNPMM will be chosen for its broad empirical basis, its global reach and coverage until 2020, and by scholars interested in the distinction between minimalist, moderate, and maximalist tasks. Moreover, the UNPMM is linked to UCDP conflict IDs and is accessible for both quantitative and qualitative researchers. PEMA, in turn, will be chosen by scholars for its coding of mandate task removal in addition to mandate task establishment, and the distinction between modalities of engagement (monitoring, assisting, securing) and between whether a task is requested or encouraged. Finally, TAMM is most adequate for researchers looking for a global dataset that also covers the Cold War period and for scholars with an interest in the distinction between peacekeeping, peacebuilding, and violence limitation tasks as well as different task orders (first and second/third-order tasks).

Regarding task lists (see Table A2 in the Annex), the UNPMM includes the following mandate tasks not covered by the other peacekeeping datasets: “elimination of chemical weapons program”, “recovery, rehabilitation, and reconstruction”, “co-ordination of donors, partners, and UN agencies”, and “conflict assessment and early warning”. It is also the only one to break Protection of Civilians (POC) into “POC general”, “POC conflict-related sexual violence”, and “POC children”. Moreover, it distinguishes between “support to permanent state institutions” and “institution-building (IB) transitional state institutions”. PEMA, in turn, is the only dataset to include “public health” and “power-sharing” as mandate tasks. In contrast to the others who only code for the “use of force” (UNPMM) or “chapter VII authorization” (TAMM), it also distinguishes between “use of force” and “offensive operations”. Both UNPMM and PEMA differentiate dialogue and reconciliation at the regional, national, and local levels. TAMM’s unique mandate tasks mostly stem from the break-up into first- and second/third-order tasks. It distinguishes for instance between “good offices” and “liaise/facilitate communication between warring parties”, between “protect UN personnel” and “protect humanitarian personnel”, or between “assist refugees” and “monitor the refugee situation”. Both PEMA and TAMM further break up DDR, PEMA into “disarmament and demobilization” and “reintegration” and TAMM into “monitor DDR” and “help implement DDR”, and both distinguish between electoral support as “electoral security”, “electoral assistance”, and “voter education” (PEMA) and “provide security during the electoral period”, “assist with the implementation of elections”, and “monitor elections” (TAMM).

## Trends in the Nature of Peace Missions

In the following, we present insights from the UNPMM data related to peace mission types, objectives, and specific tasks.^17^ To capture trends over time, we distinguish between three distinct time periods: The *post-Cold War period* (1991 to 2000) characterized by US unipolarity and the global spread of liberal democracy; the *War on Terror period* (2001 to 2010) characterized by the policy responses to the 9/11 attacks, and the *emerging multipolarity period* (2011 to 2020) characterized by a relative decline in US leadership due to the growing power and assertiveness of China, Russia, and regional powers.^18^

**Figure 1.**
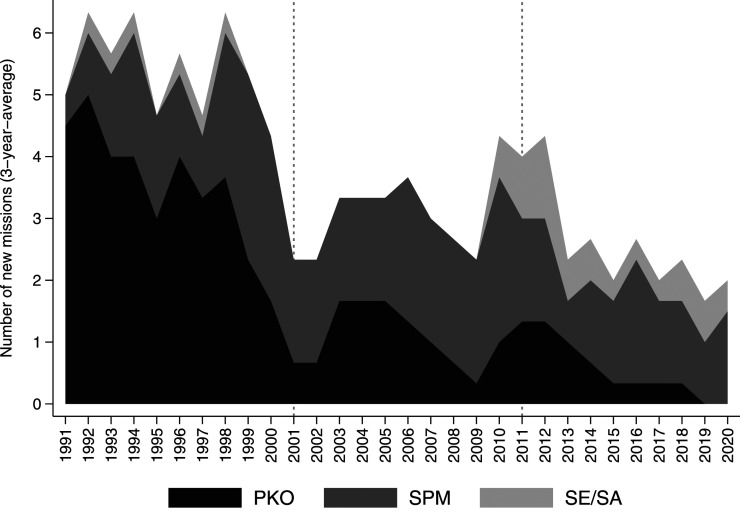
Establishment of new peace missions by mission type, 1991–2020.

Regarding mission types, our data confirms a shift away from PKOs towards political missions (Gowan 2010, Hellmüller et al. 2022). As Figure 1 illustrates, the number of newly established UN peacekeeping missions is in decline.^19^ While 36 new PKOs were established in the post-Cold War period, this number dropped to 10 in the War on Terror period. In the most recent emerging multipolarity period, only 6 new PKOs were deployed, only one of which was established in the most recent 5 years (the UN Mission for Justice Support in Haiti in 2017). The same does not apply to SPMs and SE/SAs. The number of these political missions has remained quite steady with around 20 new missions in each period, though with a relative increase of SE/SAs most recently. Echoing findings by Clayton et al. (2021), we show that the number of political missions surpasses PKOs in every year since 2000.

Behind this shift are fundamental changes on the supply side, including a larger role for regional organizations, states pressing for peacekeeping cost reductions, the Trump administration’s refusal to foot its peacekeeping bill, a UN Secretary-General with a preference for political initiatives over troop deployments, and increasing tensions among the permanent five member states (P5) in the UNSC (Boutellis and Novosseloff 2017, De Coning 2021, Coleman and Williams 2021). What is harder to gauge is the impact this shift has had on the UN’s ability to act as guarantor of international peace and security, given that most studies on peace mission impact focus on uniformed PKOs. The UNPMM points to the need to, at a minimum, account for these political missions when studying peace mission impact.

**Figure 2.**
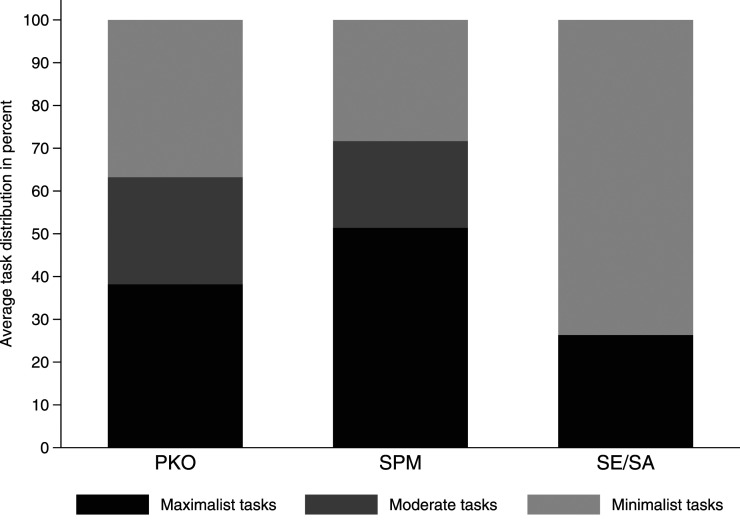
Distribution of tasks across mission types.

What we can show with our data is that the objectives and tasks differ between different types of peace missions. Figure 2 indicates that the average PKO engages across the spectrum of tasks with minimalist, moderate, and maximalist objectives. SPMs are not too different from PKOs in that regard, but usually have fewer minimalist tasks related to violence reduction, and more maximalist tasks aimed at addressing the root causes of conflict. Importantly, SE/SAs are usually appointed at the height of an ongoing conflict, for instance to work towards a ceasefire and to negotiate a political agreement, hence their tasks range more in the minimalist realm aiming at stopping the violence in the short term.

This distribution of objectives is not surprising per se. In an ideal world, the conflict context (including the phase a conflict is in) would shape what type of mission is deployed. If, however, mission type is contingent on what the UN is willing and able to commit to, as recent developments suggest, there is a mismatch between supply and demand. A closer look at the detailed mandate coding in our data drives this point home. PKOs and SPMs, in particular, are not as similar as the simple distribution of minimalist, moderate, and maximalist objectives would suggest. While both frequently engage in human rights promotion and inter-agency coordination, only PKOs are usually tasked with monitoring ceasefires and troop withdrawals, the containment of armed violence, and the protection of civilians. Thus, in the midst of active war-fighting, political missions cannot functionally substitute for a PKO.

**Figure 3.**
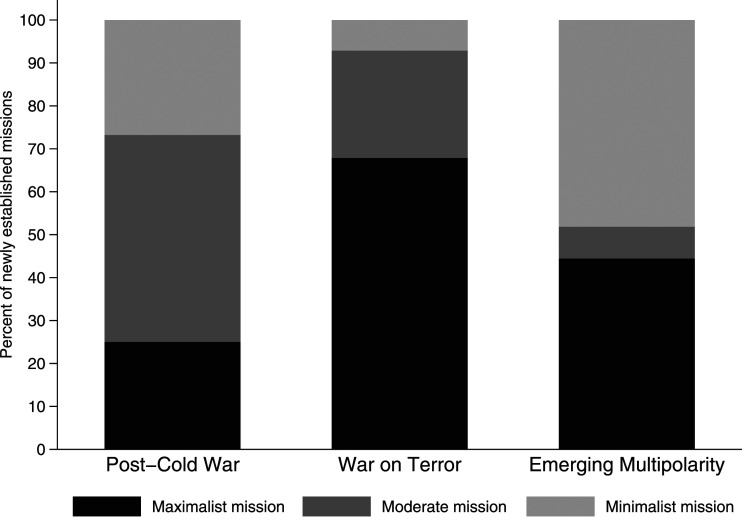
Mission objectives across time periods.

The tasks and overall objectives of UN peace missions have changed a lot over time. Overall, the UN has mandated mostly maximalist missions (45), followed by moderate (36) and minimalist (30) missions. Figure 3 shows that in the post-Cold War period, missions with moderate objectives were most frequent (27), while maximalist missions dominated during the War on Terror period (19). In the emerging multipolarity period, minimalist missions became the most commonly established mission type, though the defining feature here is polarization: New UN missions during this time were either minimalist (13) or maximalist (12), with moderate missions being rare (2), and absent in the last 5 years.

The trend in Figure 3 suggests a return to more traditional peace missions in the emerging multipolarity period. Not only is there a decrease in peace missions overall, but missions with minimalist objectives are more frequent than ever since the end of the Cold War. Moreover, there is a stark reduction in missions with maximalist objectives compared to the War on Terror period. We also see this when we look at the number of tasks missions have on average. After the Cold War, missions had an average of 7 tasks. During the War on Terror period, this number increased to 12, and then dropped to 9 in the most recent period. As UN Secretary-General Guterres put it, “Christmas is over”, in reference to the often criticized proliferation of tasks that missions were expected to fulfil during the heyday of peacekeeping (Security Council Report 2019).

There are also temporal shifts with regard to the use of force in peacekeeping mandates.^20^ On average, 40% of UN PKOs are authorized to use all necessary means – including the use of force – to enforce or implement their mandates. This number was low in the post-Cold War period (18%), and then extremely high (90%) during the War on Terror period, likely because the fight against terrorism led to a convergence in security priorities in the UNSC, making it possible to pass robust mandates for peace missions, especially in contexts that were determined as constituting a terrorist threat. The number decreased to 83% in the emerging multipolarity period, suggesting that in the few instances where the P5 could actually agree on a mission, they generally also agreed that a robust mandate was needed.

**Figure 4.**
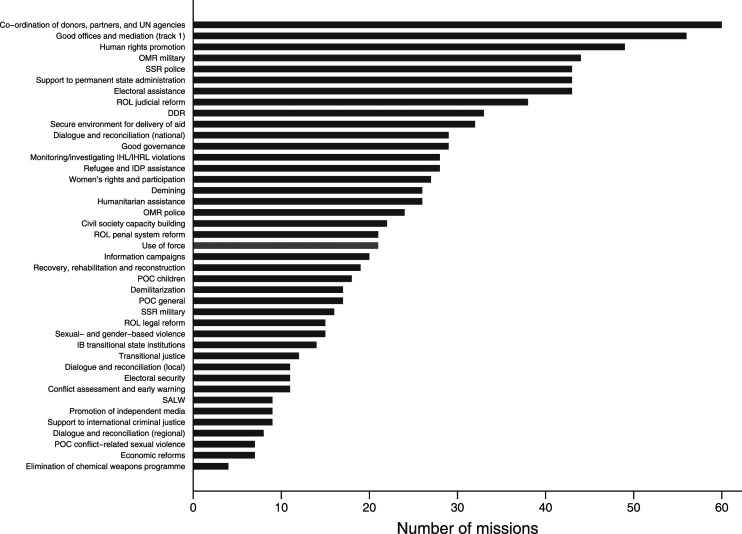
Frequency of mandate tasks across all peace missions, 1991–2020.

Our data permits analysts to identify similar trends across time for any of the 41 mandate tasks that are coded. Figure 4 lists all tasks ranked by their frequency across the entire 1991 to 2020 period. The co-ordination of donors, partners, and UN agencies stands out as the most mandated task. While the literature frequently points to the military or political capacities of UN peace missions (e.g. Tull 2009, Kjeksrud and Aasland Ravndal 2011, Berdal 2018), this observation demonstrates that coordination of actors is a core, yet underemphasized feature of UN peace missions. This opens a discussion around the central functions and value added of UN peace missions on the ground, and why these missions might prove crucial in conflict-affected contexts in spite of their purported ineffectiveness in attaining their stipulated military or political objectives.

Inter-agency coordination is followed closely by good offices and mediation as well as human rights promotion. The task most associated with traditional peacekeeping, namely the observing, monitoring, or reporting on ceasefire arrangements, withdrawals, or demilitarization, is in fourth place. Significantly, from a gender perspective, tasks related to sexual and gender-based violence rank low on the list.

**Figure 5.**
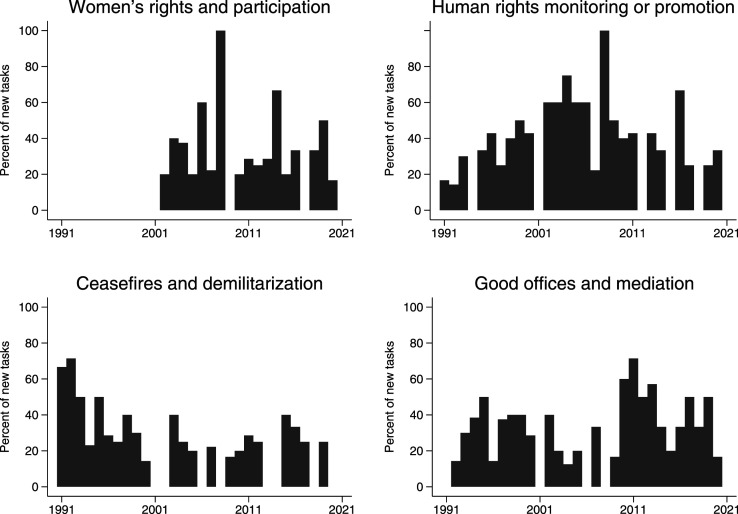
Changing frequency of tasks over time, 1991–2020.

The importance that UN member states place on certain mission tasks changes over time. Figure 5 illustrates this for four selected tasks. Specifically, it shows how often a task was mandated in a year, as a percentage of all the missions that were either established or had a mandate change in the same year. First, promoting women’s rights and participation was not a priority in peace missions in the first time period after the end of the Cold War. The flagship UNSC resolution 1325 on Women, Peace and Security adopted in 2000 changed that, but it is still a minority of peace missions that have the promotion of women’s participation in their mandate. Second, the monitoring of human rights violations and human rights promotion are some of the most frequent tasks included in peace missions overall. Yet there is a clear time trend in that this task was most common during the War on Terror period in the 2000s. Interesting is the decline in human rights mandates in the most recent period, which appears to be a consequence of a more assertive stance by Russia and China to restrain the UN’s human rights work in peace missions (Gladstone 2018). Third, there is a de-prioritization of basic military tasks, namely ceasefire monitoring and demilitarization, after the 1990s. While a relatively constant number of missions still perform these tasks, there are now more peace missions that do not have this basic military mandate. Finally, good offices and mediation are dominating in the most recent period. This is not entirely surprising, given that PKOs have often been replaced by SPMs and SE/SAs, which traditionally focus on conflict resolution.

The goal of the preliminary analysis above is to provide a more nuanced picture of the evolution of UN peace mission mandates over time than what might be gained from existing analyses, which are mostly limited to describing a shift from multidimensional to robust missions. Moreover, the extended temporal scope of our data allows us to track some of the most recent developments. It appears that an increasingly multipolar word order and related geopolitical tensions have had a fundamental impact on the number, type, objectives, and tasks of UN peace missions. To further analyze the factors that determine the kind of UN missions that are authorized, however, multivariate statistical analyses are needed. In the next section, we conduct such an analysis.

## Empirical Application: UN Peace Mission Authorization

To illustrate how the UNPMM data can be used, we replicate a study by Cordell et al. (2021) (hereafter CWD) on UN peace mission authorization, and add information on political missions and mandate tasks from the UNPMM.^21^ The study analyses factors that influence the willingness of the UN to authorize new peace operations. The authors’ argument centres on capacity and risk: Both greater existing peacekeeping commitments and perceived risks ought to reduce the UN’s propensity to create new operations. The statistical results support the arguments: First, new operations are less likely when the UN’s peacekeeping burden is already high, i.e., when the UN has more missions ongoing globally (CWD, 142–143).^22^ Second, there is a greater likelihood of a PKO after a peace agreement has been signed in the conflict, arguably because political agreements lower the risk of peacekeeping failure (CWD, 145–146). Model 1 in Table 2 shows the results of the original study.^23^

**Table 2.** Peace Mission Onset by Type, 1991 – 2016.

	(1)	(2)		(3)	
	UN PKO	UN Pol.	UN PKO	UN Pol.	UN PKO
**Global Burden & Demand**					
UN PK operations (t-1)	−0.29** (0.14)	−0.12 (0.15)	−0.25** (0.11)	0.03 (0.12)	−0.12 (0.11)
UN Pol. Operations (t-1)				0.02 (0.11)	−0.18** (0.07)
UN PK personnel (t-1)	0.00 (0.00)	0.00 (0.00)	−0.00 (0.00)	0.00 (0.00)	−0.00 (0.00)
Total civil conflicts	−0.07 (0.06)	0.03 (0.05)	−0.08 (0.06)	0.06 (0.09)	−0.20*** (0.07)
**Risk**					
Peace agreement (t-1)	1.85*** (0.51)	1.01 (0.67)	1.96*** (0.46)	1.05 (0.67)	2.03*** (0.47)
UN PK fatalities (t-1)	0.01** (0.01)	0.00 (0.01)	0.01** (0.01)	−0.00 (0.01)	0.01** (0.01)
**Conflict Characteristics**					
Civil conflict year	−0.12 (0.08)	0.01 (0.06)	−0.19** (0.08)	0.02 (0.07)	−0.19** (0.10)
Previous UN PK operation	1.16** (0.48)	2.01*** (0.66)	1.01** (0.50)	2.19*** (0.64)	1.11* (0.59)
Civilian casualties (log)	0.12** (0.06)	−0.04 (0.11)	0.16**(0.06)	−0.08 (0.12)	0.24*** (0.07)
Battle deaths (log)	0.02 (0.13)	−0.03 (0.15)	0.12 (0.11)	−0.04 (0.19)	0.12 (0.14)
**Country Characteristics**					
Population (log)	−0.35 (0.25)	−0.40* (0.23)	−0.24 (0.31)	−0.33 (0.24)	−0.19 (0.37)
Armed forces (log)	0.10 (0.21)	0.29 (0.21)	0.18 (0.25)	0.19 (0.18)	0.03 (0.28)
Democracy	−2.97*** (1.12)	1.75 (1.61)	−1.31 (1.64)	2.33 (1.66)	−0.30 (1.67)
P5 alliance	−0.05 (0.49)	−0.91 (0.62)	−0.09 (0.53)	−0.81 (0.63)	−0.14 (0.55)
Constant	7.67* (4.58)	−0.95 (4.80)	4.34 (3.95)	−4.86 (6.18)	7.17 (5.06)
*N*	*873*	*873*	*873*	*832*	*832*

*Note*: Binomial/multinomial logit. Clustered standard errors in parentheses.

*p < 0.1 **p < 0.05 ***p < 0.01.

With our detailed data on political missions and mandate tasks, we add nuance to these findings while bolstering the conclusions of the CWD study overall. Like most quantitative studies, CWD focus on peacekeeping, though their sample does include a few political missions. We add the remaining political missions (both SPMs and SE/SAs) from the UNPMM. A higher global peacekeeping burden should have less of an impact on political missions than on PKOs. This is because they are less costly and less personnel-intensive, but also because not all of these missions are authorized by the UNSC, making them less vulnerable to vetoes by powerful states. Model 2 in Table 2 shows that extant commitments in terms of ongoing PKOs indeed only reduce the likelihood of peacekeeping, but not of political missions. Peace agreements, interestingly enough, are also only correlated with peacekeeping, not political missions. One explanation for this is that a failure of these lower-profile missions is considered less costly, i.e., the UN is less risk-averse with political missions.^24^

The results in Model 2 hold when we add the political missions to the UN’s extant peacekeeping commitments. More interesting, however, is what we find when we distinguish the burden of peacekeeping and that of political missions. While one would expect the burden of political missions to be less heavy (cost, personnel, reputation, etc.), we find in Model 3 in Table 2 that more political missions make it less likely that new PKOs are authorized. This, we argue, has less to do with extant commitments and capacity than it does with general peacekeeping trends. A high number of political missions is likely an indicator of an inability of the P5 to agree on peacekeeping, as evidenced in the past few years, making the deployment of a PKO less likely. Political missions, it looks like, are used as a substitute.

In Table 3, we shift our focus from mission types to mandates. First, we make use of our classification of minimalist, moderate, and maximalist missions. If new missions are authorized under an already heavy peacekeeping burden, we should expect these to be as slim as possible, focusing only on the most crucial tasks to manage violence. And indeed, we find in Model 4 that more ongoing missions make only the creation of new maximalist missions less likely, not minimalist or moderate missions. Peace agreements, on the other hand, increase the likelihood of new missions of all ambition levels, which is solid support for CWD’s argument that UN peacekeeping authorization is influenced by the prospects of success.

**Table 3.** Peace Mission Onset by Class and Mandate, 1991 – 2016.

	(4)			(5)	
	Min.	Mod.	Max.	Noninterf.	Interfer.
**Global Burden & Demand**					
UN PK operations (t-1)	−0.03 (0.17)	−0.04 (0.17)	−0.31** (0.13)	−0.16 (0.12)	−0.17* (0.10)
UN PK personnel (t-1)	0.00 (0.00)	−0.00 (0.00)	0.00 (0.00)	0.00 (0.00)	0.00 (0.00)
Total civil conflicts	0.06 (0.11)	0.06 (0.07)	−0.10* (0.06)	0.02 (0.06)	−0.11* (0.06)
**Risk**					
Peace agreement (t-1)	1.55** (0.75)	2.41*** (0.69)	1.23** (0.50)	1.76*** (0.44)	1.40*** (0.49)
UN PK fatalities (t-1)	−0.01 (0.01)	0.01 (0.01)	0.01 (0.01)	0.01 (0.01)	0.01 (0.01)
**Conflict Characteristics**					
Civil conflict year	−0.07 (0.14)	−0.12 (0.18)	−0.07 (0.06)	−0.03 (0.08)	−0.10 (0.07)
Previous UN PK operation	0.09 (1.41)	1.11 (0.74)	1.79*** (0.45)	1.11** (0.57)	1.86*** (0.59)
Civilian casualties (log)	0.17 (0.15)	−0.06 (0.07)	0.17* (0.09)	0.02 (0.07)	0.22** (0.10)
Battle deaths (log)	0.13 (0.38)	0.03 (0.20)	0.01 (0.11)	0.07 (0.16)	−0.01 (0.14)
**Country Characteristics**					
Population (log)	−1.31*** (0.41)	−0.35(0.44)	0.06 (0.35)	−0.57** (0.26)	0.01 (0.34)
Armed forces (log)	1.18* (0.63)	0.25 (0.37)	−0.09 (0.22)	0.37 (0.24)	−0.04 (0.22)
Democracy	0.76 (1.26)	−2.88 (2.34)	0.34 (1.65)	−1.12 (1.12)	1.49 (1.64)
P5 alliance	−0.15 (0.90)	−0.10 (0.75)	−0.52 (0.61)	−0.02 (0.56)	−1.14**(0.50)
Constant	0.13 (6.80)	−2.49 (5.45)	3.18 (4.67)	3.02 (3.96)	0.79 (5.17)
*N*	*873*	*873*	*873*	*873*	*873*

*Note*: Binomial/multinomial logit. Clustered standard errors in parentheses.

*p < 0.1 **p < 0.05 ***p < 0.01.

In our final model, we illustrate how future research can make use of the detailed coding of mandate tasks in the UNPMM. In all their models, CWD include the control variable P5 Alliance, which records whether the conflict country has a formal military alliance with one of the five permanent members of the UNSC. They argue that PKOs should be less likely in countries with such an alliance, given that the major powers can block peacekeeping in states with whom they have a strategic relationship. The question is whether and why the P5 would always *want* to block peacekeeping in their alliance partners’ conflicts? In fact, Allen and Yuen (2014) show that it is P5 apathy towards a conflict country, rather than P5 interest, that restricts UN peacekeeping in these conflicts, and indeed, the variable is not significant in CWD’s models.^25^

We argue instead that an alliance with a P5 does not make PKOs per se less likely, but rather missions of certain types. Specifically, we posit that a P5 member would block missions that have a mandate to meddle in what the alliance partner considers their internal affairs. To test this, we created a variable that records whether a peace mission has a mandate for civil society capacity building, ensuring good governance, or promoting free media. Authoritarian states in particular are sensitive to missions that interfere with these civil liberty-related issues and may lobby against the inclusion of such tasks with their P5 partner. And indeed, we find in Model 5 in Table 3 that in conflict countries that have a P5 alliance, only the creation of such “interfering” missions is less likely, not missions in general. The same is true, even though not reported in the Tables here, for missions that have a mandate for Security Sector Reform (SSR). We expected the P5 to be wary of letting peace missions interfere with their alliance partners’ military affairs, and that is exactly what we find.

Taken together, the findings presented here offer support to CWD’s argument that capacities and perceived risks (or the prospect of success) play an important role in the UN’s decision to authorize new peace operations, but that not all types of peace missions are equally sensitive to these calculations. Specifically, political missions and missions with more limited ambitions do not seem to be affected by extant peacekeeping commitments. The same appears to be true for powerful states’ relationships with the conflict country. They matter, but less for the decision of whether to authorize a mission than for the type of mandate the UNSC members are willing to agree to. This latter finding in particular shows how future research can make use of our detailed mandate coding to study the influence of powerful states over the shape of peace missions in a more nuanced manner (see also Allen and Yuen 2014).

## Conclusion

The UNPMM dataset introduced in this article serves to study the evolving nature of UN peace missions. With global coverage, 30 years of data between 1991 and 2020, a broad scope that includes not only peacekeeping but also political missions, and detailed information on 41 mandate tasks, the UNPMM currently presents one of the most detailed and up-to-date global datasets on UN peace mission mandates. Moreover, even scholars not needing this level of granularity in terms of mandate tasks can draw on our novel categorization that distinguishes missions with minimalist, moderate, and maximalist objectives – a distinction that builds on the idea of negative and positive peace familiar to all peace researchers.

Looking ahead, the UNPMM can be used to study the nature of peace missions from various angles. First, scholars can deepen the type of analysis we have conducted in our empirical example and use the UNPMM data as an *explanandum* – an outcome to be explained. To give just a few examples, the UNPMM can be used to address questions such as: How do particular characteristics of a conflict, the regional and international security environment, or global priorities and geopolitics influence what tasks missions receive? What shapes whether missions receive a mandate to protect children specifically, address sexual violence, or conduct local dialogue and reconciliation? Finally, what role do rising powers play as troop contributors and what is their impact on UN peace mission mandates (Fung 2015, Abdenur and Call 2017, Sazak and Woods 2017)?

Second, the data can be used to add nuance to studies of peace mission effectiveness. Here, the UNPMM data is used as an *explanans* to help explain different outcomes in conflict contexts. Quantitative studies usually define successful peacekeeping in terms of a mission’s impact on reducing violence (Fortna 2008, Dorussen 2014, Di Salvatore and Ruggeri 2017, Kathman and Benson 2019) and on the duration of peace (Fortna 2004, Gilligan and Sergenti 2008, Joshi 2013). Qualitative scholars provide more granular assessments of effectiveness, going beyond negative peace and including broader factors such as structural inequalities, respect for women’s rights, and aspects related to a functioning state (Van der Lijn 2009, Olsson 2009, Olsson and Gizelis 2014). The UNPMM can advance this research by allowing for a measurement of effectiveness against a mission’s mandated tasks rather than broader conceptions of non-violence (Tull 2009, Duursma and Svensson 2019). Research can for instance focus on the achievement of mandate tasks such as POC (Hultman 2013, Hultman et al. 2013, Bove and Ruggeri 2016, Fjelde et al. 2018, Johansson and Hultman 2019), electoral security (Smidt 2020), or state-building tasks (Roberts 2008, De Heredia 2012). Moreover, the UNPMM enables scholars to move beyond an evaluation of peace missions that focus on single mandate activities and look at the interactive effects that several, all, or a specific category of mandate tasks might have on its effectiveness (Caplan 2020, 71).

Third, the UNPMM contributes to research on local-global interactions in two ways. First, current research entails a strong critique of liberal peacebuilding and a call for a reconceptualization of the “local” in UN peace missions (Autesserre 2010, Tadjbakhsh 2011). Yet, scholars also point to the risk of using strict binaries of local-international or liberal-illiberal, pointing to the fluidity of these terms (Gamez 2021, Millar 2021, Hellmüller 2021b). The UNPMM contributes to these developments by purposefully applying the more openly phrased minimalist-moderate-maximalist categorization (Call and Cousens 2007). Thereby, the data can be used to examine UN peace missions based on an open typology that nevertheless captures important qualitative information about the objectives of each particular mission. Second, the UNPMM allows for an analysis of how UN peace missions are adapted to local priorities. UNPMM data can be combined with studies on local perceptions of peace. For instance, the Everyday Peace Indicators (EPI) developed by Firchow and Mac Ginty (2017) could be compared to the UNPMM data on peace missions’ mandated tasks to examine the extent to which they correspond (or not).

Overall, the UNPMM provides the data needed to understand the evolving nature of UN peace missions. The trends and statistical analysis presented in this paper point to the need for a greater disaggregation of data on UN peace missions and their mandates. This has become especially important in current times as we bear witness to tectonic shifts in world politics and, as a result, in the role of the UN in maintaining international peace and security.

## Supplemental Material

Supplemental Material - What Is in a Mandate? Introducing the UN Peace Mission Mandates Dataset Click here for additional data file. Supplemental Material for What Is in a Mandate? Introducing the UN Peace Mission Mandates Dataset by Sara Hellmüller, Xiang-Yun Rosalind Tan, and Corinne Bara in Journal of Conflict Resolution

Supplemental Material - What Is in a Mandate? Introducing the UN Peace Mission Mandates Dataset Click here for additional data file. Supplemental Material for What Is in a Mandate? Introducing the UN Peace Mission Mandates Dataset by Sara Hellmüller, Xiang-Yun Rosalind Tan, and Corinne Bara in Journal of Conflict Resolution

Supplemental Material - What Is in a Mandate? Introducing the UN Peace Mission Mandates Dataset Click here for additional data file. Supplemental Material for What Is in a Mandate? Introducing the UN Peace Mission Mandates Dataset by Sara Hellmüller, Xiang-Yun Rosalind Tan, and Corinne Bara in Journal of Conflict Resolution

## Notes

1. The term “UN Peace Missions” in this paper entails UN Peacekeeping Operations (PKOs), Special Political Missions (SPMs), as well as appointments of Special Envoys/Advisers (SE/SAs).

2. The descriptive statistics in this paper make use of Version 1.5 of the UNPMM data, available in the supplementary materials and at www.peacemissions.info.

3. In addition to the 40 mandate tasks, the dataset also codes what other organizations UN missions are mandated to cooperate with, which then brings the total number to 50 tasks.

4. There are also institutional and budgetary differences, in that political missions are managed by the UN Department of Political and Peacebuilding Affairs and funded through the regular budget, while PKOs are led by the UN Department of Peace Operations and funded under both the regular budget as well as a mission-specific one.

5. These terms were first suggested by Call and Cousens (2007) as a way to measure successful peacebuilding outcomes.

6. As opposed to 2015 for TAMM and UNPI, and 2017 for PEMA. Since 2017, there have been missions in Colombia, Haiti, Iraq, Mozambique, Myanmar, and Yemen.

7. As compared to 27 of PEMA and 72 of TAMM. UNPI has 469, as it coded all UN peace initiatives.

8. As opposed to 27 for UNPI, 40 for TAMM, and 41 for PEMA.

9. Europe: 82% (PKO) vs 18% (SPMs and SE/SAs); Africa: 50% vs 50%; Americas: 50% vs 50%; Asia: 35% vs 65%; Middle East: 15% vs 85%. These calculations are based on the 111 fully coded missions only.

10. Africa: 46% (maximalist) vs 33% (moderate) vs 21% (minimalist); Americas: 50% vs 31% vs 19%; Asia: 47% vs 47% vs 6%; Middle East: 15% vs 8% vs 77%; Europe: 18% vs 36% vs 46%.

11. 1991–2000: 25% maximalist vs 48% (moderate) vs 27% (minimalist); 2001–2010: 68% vs 25% vs 7%; 2011–2020: 45% vs 7% vs 48%.

12. A total of 111 missions were established between 1991 and 2020. Two more missions started before 1991 but had mandate changes after 1991: the UN Peacekeeping Force in Cyprus (UNFICYP) and the UN Interim Force in Lebanon (UNIFIL). The post-1991 mandate changes of these missions are coded in the dataset, but these missions are not assigned mission objective scores. 9 other missions that were established before 1991 but active as of 1 January 1991 are included in the dataset for completeness, but as there were no mandate changes post-1991, no mandate tasks are coded for these missions.

13. See codebook for detailed descriptions of mandate tasks.

14. Institution-building (IB) transitional state institutions includes instances where the UN takes over the state administration and acts as the transitional authority, such as in the UN Mission in Kosovo (UNMIK), the UN Integrated Mission in Timor-Leste (UNMIT), or the UN Transitional Authority in Cambodia (UNTAC).

15. Small arms and light weapons (SALW) concerns monitoring the illicit flow of small arms and light weapons. The term “SALW” needed to be explicitly mentioned in a mission’s mandate for this task to be coded.

16. Use of force is an overarching category (ocat) (neither minimalist, moderate, or maximalist).

17. Code to replicate these statistics is in the supplementary material.

18. This partitioning with the data is in line with Howard and Stark (2017/18) who divide the post-Cold War period into a post-Cold War democratization phase dominated by US unipolarity and liberal democracy from 1991 to 2000 and a post-September 11 phase characterized by the War on Terror. We added a third phase of emerging multipolarity as many authors locate the rise of multipolarity around 2010 (Harland 2016, Ikenberry 2018, Paul 2018).

19. Note, however, that the number of deployed UN troops, police, and observers globally has been steadily increasing until very recently (see Bara and Hultman 2020, Coleman and Williams 2021).

20. We do not count political missions here, as they are never allowed to use force given their purely civilian make-up.

21. Replication data and code for this analysis are in the supplementary material. Note: This replication was conducted with UNPMM V1.4. However, the differences between V1.4 and V1.5 are minor and do not involve any changes to mission type and class that are used in this analysis.

22. The authors use multiple indicators for commitments and risk, but the others are not statistically significant.

23. We make two changes to the original analysis, which do not change CWD’s findings. First, we drop the indicator for peacekeeping expenditures from the models, as it is strongly correlated with personnel commitments. Second, we use standard errors clustered on countries.

24. The finding could also be due to the way the CWD data is set up. Countries exit the sample after the onset of a PKO. We can thus only predict the onset of political missions *before* or *instead* of PKOs, and these tend to have a mandate to work *towards* a peace agreement. This would explain why their onset is not correlated with agreements already reached.

25. One should be careful when interpreting control variables (see Cinelli and Hazlett 2020). They are included to get unbiased estimates for the main variables of interest, and if we want an unbiased estimate of the P5 Alliance variable, we need to control for factors that confound the relationship between this particular variable and the outcome. The findings presented in Model 5 should thus be taken with a grain of salt. They point to a promising area for further research and illustrate how this research, with properly specified models, can make use of the detailed UNPMM mandate coding.
