# Supplementary material for: What is in a Mandate? Introducing the UN Peace Mission Mandates Dataset
Source: J Conflict Resolut. 2023 Mar 2;68(1):166–92. doi: 10.1177/00220027231159830 (PMC10663125; doi:10.1177/00220027231159830)
Supplement: Supplemental Material - What Is in a Mandate? Introducing the UN Peace Mission Mandates Dataset [file sj-pdf-3-jcr-10.1177_00220027231159830.pdf]

Annex

Table A1. Comparison of main features in datasets on peace mission mandates.

	**UNPMM**	**PEMA**	**TAMM (2.1)**	**UNPI**
**EMPIRICAL BASIS**	112 peace missions (from which 54 PKOs)	27 PKOs	72 PKOs	469 peace initiatives
**NUMBER OF CODED TASKS**	41	41	40	27
**REGIONAL SCOPE**	Global	Africa	Global	Global
**TEMPORAL SCOPE**	1991-2020	1991-2017	1948-2015	1946-2015
**LINKED DATASETS**	UCDP/PRIO Armed Conflict Dataset (ACD)	N/A	N/A	UCDP/PRIO Armed Conflict Dataset (ACD)
**FURTHER DISTINCTIONS**	Objectives:<br>Minimalist,<br>Moderate,<br>Maximalist	Modality of engagement:<br>Monitoring,<br>Assisting,<br>Securing<br><br>Task:<br>Requested,<br>Encouraged	Function:<br>Peacekeeping,<br>Peacebuilding,<br>Violence limitation<br><br>Task order:<br>First-order,<br>Second-order,<br>Third-order	Establishment and authorization:<br>Security Council,<br>Secretary-General,<br>General Assembly<br><br>Thematic cluster:<br>Diplomatic,<br>Technocratic,<br>Political, Development,<br>Peacekeeping
**ADDITIONAL FEATURES**	Data presented in raw format for statistical analysis as well as a user-friendly excel sheet	Codes for removal of mandate tasks		

Table A2. Overview of task lists contained in datasets on peace mission mandates.

	UNPMM	PEMA	TAMM (2.1)	UNPI
Humanitarian	refugee / IDP assistance	refugee assistance	assist refugees	facilitate return of refugees
	humanitarian assistance	humanitarian relief	facilitate the delivery of humanitarian assistance	
			protect humanitarian personnel	
			protect UN personnel	
	secure environment for delivery of aid		assist implementing quick impact projects (incl. through provision of a safe environment)	
Security	Demining	demining	assist demining	demining
	DDR	disarmament and demobilization	monitor DDR	DDR
		reintegration	help implement DDR	
	OMR military	ceasefire assistance	monitor a ceasefire or peace agreement	monitoring
	OMR police	border control	monitor a buffer zone	promote cross-border cooperation
			help implement a ceasefire or peace agreement	
			monitor host country's borders	
		arms embargo assistance	monitor weapon's trade	
			monitor weapon's embargo	
			inspect cargo	
	SSR military	military reform	assist security sector reform	security sector reform
	SSR police	police reform	assist police reform	
			monitor the police	
			peacekeepers conduct joint patrols with police	
	POC general	civilian protection	protection of civilians	protection of civilians
	POC conflict-related sexual violence		prevent rape/protect women	
	POC children			address the needs of children in peacebuilding
	SALW	SALW		

	Demilitarization	demilitarization		
	sexual- and gender-based violence	SGB violence		
	elimination of chemical weapons program			
**Economic**	economic reforms	economic development		economic development
		resources	monitor use of natural resources	
		cultural heritage	assist in the prevention of attacks on cultural and historical sites	
	recovery, rehabilitation, and reconstruction			
**Political**	information campaigns	public information	disseminate information about the mission to the public	education
	electoral security	electoral security	provide security during the electoral period	election support
	electoral assistance	electoral assistance	monitor elections	
		voter education	assist with the implementation of elections	
	good offices and mediation	peace process	establish or exercise good offices	dialogue assistance
			liaise / facilitate communication between warring parties	
	women's rights and participation	gender mainstreaming		address issues of gender
	support to permanent state institutions	political party assistance	help build government capacity	founding a new state, including its institutions
	IB transitional state institutions	state authority assistance	help implement government policies	
	dialogue and reconciliation (national)	national reconciliation	promote national reconciliation	
	dialogue and reconciliation (regional)	regional reconciliation		
	dialogue and reconciliation (local)	local reconciliation		
	good governance	democratization		governance
	promotion of independent media	media assistance	promote freedom of press	
	civil society capacity building	civil society assistance		
	co-ordination of donors, partners, and UN agencies			coordinate international activities
				advice

	conflict assessment and early warning			
				decolonization assistance
		public health		
		power sharing		
**Legal**	human rights promotion	human rights	protect human rights	human rights promotion
	monitoring / investigating IHL/IHRL violations	child rights	protect children	fact-finding
			monitoring human rights	
			monitor the refugee situation	
	ROL penal system reform	prison reform		promote rule of law
	ROL legal reform	legal reform		
	ROL judicial reform	justice sector reform	assist with justice sector reform	
	transitional justice	transitional justice		truth and reconciliation / transitional justice
	support to international criminal justice		pursue justice for war criminals	arbitration
**Use of force**	use of force	use of force	Chapter VII authorization	
		offensive operations		
**Other**				peacekeeping operations
				implementation
				peacebuilding misc.
				preparatory missions
